# Supplementary material for: Are carbon emissions trading and green financial instruments synergistic? -Comprehensive quantitative research based on content analysis
Source: PLoS One. 2024 Mar 7;19(3):e0298601. doi: 10.1371/journal.pone.0298601 (PMC10919601; doi:10.1371/journal.pone.0298601)
Supplement: S1 Data — (ZIP) [file pone.0298601.s001.zip › Data/地级市Z_F工作报告.zip!/╔╜╬≈/╠1⁄2╘¡╩╨/└┤╫╘╣1⁄2╓┌║┼í╛┬φ┐╦╩2╛▌═°í┐_2016.docx]

各位代表：

现在，我代表市人民政府向大会报告工作，请予审议，并请市政协委员和其他列席人员提出意见。

　　一、“十二五”时期及2015年经济社会发展回顾

　　“十二五”时期是我市发展很不平凡的五年。五年来，我们认真贯彻落实党的十八大、十八届三中、四中、五中全会精神和习近平总书记系列重要讲话精神，坚持稳中求进工作总基调，积极适应和引领经济发展新常态，全市经济社会发展取得新成就。特别是2014年9月以来，在以王儒林书记为班长的新的省委和市委的坚强领导下，全市上下按照“四个全面”战略布局，坚持“深入学习贯彻习近平总书记系列重要讲话精神，净化政治生态、实现弊革风清、重塑山西形象、促进富民强省”五句话的总要求和总思路，始终保持惩治腐败、狠刹四风、打黑除恶“三个高压态势”，以“五个一批”为载体，推动“六大发展”，实施“三个突破”，努力在全省发挥“六个表率”作用，开创了各项事业发展的新局面。全市地区生产总值由2010年的1781亿元增加到2015年的 2735.34亿元，年均增长8.4%；固定资产投资由2010年的916.48亿元增加到 2025.61亿元，年均增长18.2%；社会消费品零售总额由2010年的825.85亿元增加到1540.8亿元，年均增长13.3%；一般公共预算收入由2010年的138.48亿元增加到274.24亿元，年均增长14.6%；城镇常住居民人均可支配收入达到27727元，农村常住居民人均可支配收入达到13626元，两项收入增速均高于地区生产总值增速，为“十三五”发展奠定了坚实基础。

2015年，面对经济下行压力，全市上下积极作为、奋力拼搏，着力稳增长、促改革、调结构、惠民生、防风险，保持了经济社会平稳健康发展。地区生产总值增长8.9%，规模以上工业增加值增长5.7%，固定资产投资增长16%，社会消费品零售总额增长6.2%，一般公共预算收入增长5.9%，城镇常住居民人均可支配收入增长7.6%，农村常住居民人均可支配收入增长8%，综合实力进一步提升。

——大力推进产业结构调整，服务业对地区生产总值的贡献率明显提升。培育发展高端装备制造、新能源、新材料、节能环保、食品药品等新兴产业，推进开发区扩区拓展，招商引资力度进一步加大。阳煤化工、江铃重汽、华润万象城、欧亚锦绣城市综合体、宝迪屠宰加工、润恒冷链物流等重点项目进展顺利。新兴接替产业增加值占到规模以上工业的67.1%，装备制造业增加值占到规模以上工业的44.6%。服务业投资占到全市固定资产投资的75.8%，增加值占到地区生产总值的61.3%。

——持续推进城市基础设施建设，城市承载力和发展水平进一步提高。新改建主次干道31项，改造背街小巷32条，建设里程113.23公里。太榆路、学府街、南内环街、南沙河路等相继改造完工，城市快速路网体系日趋完善，逐步进入立体交通时代。地铁2号线一期全线招标开工，首开段车站主体工程封顶。新改建供水管网232公里、供气管网178公里。500千伏等9项供电工程竣工投运。加大历史文化名城保护力度，推进青龙古镇、明太原县城等农耕文明保护。开工建设晋阳湖、和平公园等13个公园，新建46个小游园，完成阳兴大道、建设路、南沙河路等主干道景观绿化，新增绿地3000亩，建成区绿化覆盖率、绿地率、人均公园绿地面积分别达到41%、36.07%、11.56平方米。

——举全市之力推进城中村改造，人民生活环境进一步改善。推动54个城中村改造，47个村基本完成整村拆除，完成总拆迁量的88%，46个村启动安置房建设。城中村改造取得重要突破，省委王儒林书记要求全省学习我市城中村改造所体现出的“积极作为、攻坚克难、依法办事、为民谋利”的精神。推进棚户区改造，新开工保障性住房57711套，基本建成45510套，完成投资118.65亿元。

——全面推进“五大工程”“五项整治”，省城环境质量进一步好转。市区空气质量综合指数下降7.76%，优良天数达到230天、比上年增加33天，优良率达到63%、比上年提高9个百分点，6项主要污染物排放量均好于省下达的减排要求。集中供热扩网3104万平方米，实施城边村气化改造16个，减少冬季燃煤100万吨。关停二电厂3×20万千瓦燃煤机组等污染企业34家，减少燃煤180万吨。城南污水处理厂新增日处理能力15万吨，晋阳污水处理厂通水调试。淘汰老旧机动车和黄标车3.35万辆。秸秆综合利用80.92万亩。完成营造林29.07万亩，森林覆盖率达到23%。

——不断加大民生保障和改善力度，人民群众幸福感、获得感进一步增强。提升托底保障能力，民生支出346.7亿元，占一般公共预算支出的82.5%。城镇新增就业10.5万人。实现脱贫1.67万人。扎实推进教育卫生事业，16所新续建学校、12所改扩建医院进展顺利，16所村办幼儿园主体完工。办好一批民生实事，完成既有建筑节能改造412万平方米，新建公共停车位8160个，解决2.28万农村人口饮水安全。采煤沉陷区治理取得阶段性成果。举办太原国际马拉松赛，推进汾河体育健身长廊建设，我市体育健儿在全运会、青运会、省运会等重大赛事取得优异成绩。高度重视安全生产，安全生产事故和死亡人数分别下降4.46%、3.25%。推进“平安省城”建设，严厉打击各类违法犯罪，社会保持和谐稳定。

——全面实施“三个突破”，发展动力和活力进一步提升。加大科技创新力度，全社会研究试验经费投入93.21亿元，新增国家重点实验室1个，新增高新技术企业107家、增长39.77%，市内技术合同成交额21.96亿元、增长162%，高新技术企业销售额占到规模以上工业企业的29%。加快金融改革创新，“新三板”挂牌企业达到20家，占到全省的三分之二。推进民营经济发展，获得全国首批小微企业创业创新基地城市示范，新增民营企业1.88万户、增长16.22%，实现民营经济增加值 1571.53亿元、增长10.1%。

——深入推进“六权治本”，政府自身建设进一步加强。严格落实“两个责任”，狠刹“四风”，严肃问责不作为、慢作为。编制完成市级权力清单和责任清单，行政职权事项由6033项精简到2764项，精简率达到54%。取消、调整、下放行政审批事项119项，审批时限压缩21.6%。公务用车改革有序推进，涉改公车全部封停。全面实行政务公开、企务公开和村务公开，建立全方位、全过程、多层次的权力制约监督机制。自觉接受人大、政协监督，认真听取各民主党派、工商联、无党派人士建言献策。办理人大代表建议和政协提案851件，市人大常委会审议意见22件。深入开展“三严三实”专题教育，干部作风明显转变，干事创业氛围更加浓厚。

各位代表，五年的成绩来之不易。这是我们坚决贯彻落实党中央决策部署和省委一系列治晋兴晋强晋战略举措的结果，是省委、省政府和市委统揽全局、正确领导，市人大、市政协有效监督、全力支持，全市人民同心同德、团结奋斗的结果。在此，我代表市人民政府，向全市人民，向驻并解放军、武警官兵和中央、省驻并单位，向尽心履职的各位人大代表、政协委员、各民主党派、各人民团体，向所有关心、支持、参与太原改革发展的社会各界人士，表示崇高的敬意和衷心的感谢！

回顾过去的五年，我们也清醒地看到经济社会发展中仍然存在一些困难和问题。经济结构不优、效益不高的问题仍然突出，传统产业产能过剩、亏损严重；园区承载能力、引领作用仍然不强，好项目、大项目不多的老问题没有有效破解；基础设施、民生改善、环境治理、脱贫攻坚等短板仍然明显，兜底保障能力有待提升；管理创新仍然不够，社会治理还存在不少薄弱环节；安全生产形势仍然严峻，社会潜在风险较多；政府自身建设仍有差距，职能转变仍显滞后，“四风”问题尚未根除。对此，我们将积极面对、勇于担当、认真解决。

　　二、“十三五”时期经济社会发展的指导思想和目标任务

“十三五”时期是全面建成小康社会的决胜阶段。根据市委十届七次全会精神及“十三五”规划《建议》，“十三五”时期我市经济社会发展的指导思想是：高举中国特色社会主义伟大旗帜，全面贯彻党的十八大和十八届三中、四中、五中全会精神，坚持以马克思列宁主义、毛泽东思想、邓小平理论、“三个代表”重要思想、科学发展观为指导，深入贯彻习近平总书记系列重要讲话精神，协调推进“四个全面”战略布局，坚持发展是第一要务，树立五大发展理念，落实省委“五句话”总要求，以实施“五个一批”为重要载体和抓手，积极推进创新发展、协调发展、绿色发展、开放发展、共享发展、廉洁和安全发展，着力提高发展质量和效益，着力保障和改善民生，统筹推进经济建设、政治建设、文化建设、社会建设、生态文明建设和党的建设，努力实现在全省全面建成小康社会进程中率先发展。

　　“十三五”时期经济社会发展的主要目标是：经济结构得到新优化。地区生产总值年均增长7.5%以上，力争提前一年实现翻番、到2020年经济总量突破4000亿元。民生改善达到新水平。居民人均收入提前一年比2010年翻一番，力争到2020年城镇常住居民人均可支配收入达到4万元、农村常住居民人均可支配收入达到2万元；力争提前两年实现贫困人口脱贫、贫困县摘帽。城市功能实现新提升。城市空间布局更加优化，基础设施不断完善；城中村和棚户区改造全部完成，省会城市功能和辐射引领作用不断增强。文化建设取得新进步。人民群众思想道德、文化素质、健康水平明显提高，力争进入全国文明城市行列；国家历史文化名城影响力进一步扩大。生态环境得到新改善。主要污染物排放量、 PM 2.5浓度逐年下降，重污染天气大幅减少，市区优良天气率力争达到80%左右，森林覆盖率力争达到30%，建成区绿化覆盖率达到42%以上，城乡人居环境全面改善。改革开放实现新突破。资源型经济转型综合配套改革试验区建设取得重要进展，重点领域和关键环节改革取得决定性成果，发展动力和活力显著增强，开放型经济和对外合作机制基本形成。民主法治取得新进展。人民民主不断扩大，法治政府基本建成，司法公信力明显提高，人民权益切实保障；法治太原建设全面推进，社会治理能力和水平不断提高，社会更加和谐稳定。

“十三五”规划《纲要（草案）》已印发各位代表，这里对主要任务作简要报告。

（一）推动创新发展，着力提高发展质量和效益。深化供给侧结构性改革，大力发展高端制造业和现代服务业，培育壮大都市现代农业。充分发挥科技创新的引领作用，推动民营经济做大做强，把太原建成具有较强影响力的金融聚集区。

（二）推动协调发展，着力形成均衡发展结构。围绕改善民生，促进经济社会协调发展、城乡协调发展、物质文明和精神文明协调发展。以迎接2019年第二届全国青年运动会为契机，推进“五城联创”。促进军民融合深度发展。

（三）推动绿色发展，着力改善生态环境质量。落实主体功能区规划。实行最严格的环境保护制度，实施大气、水、土壤污染防治行动计划，深入推进省城环境质量改善。实施城市周边百万亩森林围城工程和以汾河为重点的生态环境修复工程，促进人与自然和谐共生。

（四）推动开放发展，着力实现合作共赢。建立健全对外开放政策机制，加快开发区和开放平台建设，不断拓展对外交流合作新领域。积极参与国家“一带一路”建设，推进与京津冀、环渤海经济圈协同发展，深化太原城市群协作，努力形成全面对外开放新格局。

（五）推动共享发展，着力保障和改善民生。增加公共服务供给，推进城乡基本服务均等化。办好一批民生实事。全面打赢三年脱贫攻坚战。统筹人口均衡发展，提高教育医疗水平。促进就业创业，提高城乡居民收入，建立公平、可持续、全覆盖的社会保障制度。

（六）推动廉洁和安全发展，着力夯实发展基础。全面推进“六权治本”，严格落实“两个责任”，营造廉洁发展社会环境。强化安全红线意识，夯实安全生产基础。全面加强社会治安综合治理，深入推进“平安省城”建设。

　　三、2016年工作安排

2016年是“十三五”开局之年，也是推进供给侧结构性改革的攻坚之年。我们要主动适应经济发展新常态，认真落实省委提出的“六个表率”作用的要求，用好用足省政府出台的《关于支持太原率先发展的意见》，坚定信心、抢抓机遇、奋力拼搏，在全省全面建成小康社会进程中率先发展，努力实现“十三五”良好开局。

经济社会发展的主要预期目标是：地区生产总值增长8%左右，固定资产投资增长12%，一般公共预算收入增长3%，社会消费品零售总额增长6.5%，城镇和农村常住居民人均可支配收入分别增长8%和8%以上，居民消费价格涨幅控制在3%，城镇新增就业人数8.5万人，城镇登记失业率控制在4%以内。

约束性指标是：万元地区生产总值能耗、二氧化碳排放量、用水量，主要污染物减排，市区空气质量优良天数比例，劣Ⅴ类水体比例，新增建设用地降幅，农村贫困人口脱贫人数，城市棚户区住房改造数量，均完成省下达任务。

围绕上述目标，在全面推进经济社会发展的同时，重点抓好以下八个方面的工作：

（一）深化供给侧结构性改革，推动产业转型升级。当前，我国经济发展进入速度换档、结构调整、动力转换节点，我们必须把发展基点放在创新上，把改善供给结构作为主攻方向，以培育战略性新兴产业和现代服务业为抓手，提高供给结构对需求变化的适应性和灵活性，提高供给体系质量和效率，提高投资有效性，加快培育形成新的增长动力。

做好供给侧结构性改革的“加法”，瞄准世界产业技术发展前沿，招商引资、招才引智，加大对人力资本的可持续投入，倾力支持创新型企业发展。抓好富士康手机制造和维保、太重风电装备、江铃重汽、比亚迪新能源汽车、太钢碳纤维 T800、 纳克润滑油、阳煤化工等一批高端制造业项目；抓好华润万象城、欧亚锦绣、万达综合体、苏宁电器、远大购物广场、传化物流等一批现代服务业项目；围绕低碳发展为主题的太原论坛等，打造国际化会展平台，大力发展会展经济，在更高水平上实现可持续发展。做好供给侧结构性改革的“减法”，开展“降低实体经济企业成本行动”，落实好中央和省出台的企业减负松绑政策措施，为企业降低税费、财务、物流、人工、制度性交易等成本，用新技术改造、提升、优化煤焦冶电等传统产业，促进经济持续增长。做好供给侧结构性改革的“乘法”，实施创新驱动战略，推动“双创”和“中国制造2025”“互联网+”行动计划，发挥企业在创新中的主体作用，以新供给创造新需求，以新技术带动新产业，以新空间发展新业态，培育经济增长的“乘数因子”，创造新产业“几何式增长”。做好供给侧结构性改革的“除法”，有效化解过剩产能，化解房地产库存，清理“僵尸企业”，促进产业优化重组，提升要素投入的综合效率，清除经济发展路上的“拦路虎”。不断破解制约发展的一切束缚，激发全社会的创造力，让新动能茁壮成长，传统动能焕发生机。

（二）继续推进城市基础设施建设，不断提升城市品质和服务功能。太原“三面环山、一水中分”，具有好山好水好风光自然之美。要尊重城市发展规律，紧紧围绕建设生态宜居城市的目标，更加注重城市基础设施功能完善，更加注重城市建设管理质量，更加注重地上地下统筹。

加快完善快速路、主次干路和支路级配合理的路网体系，提高道路通达性和出行便利性。新建滨河西路南延、卧虎山路，续建太行路南延、龙城大街东延、南内环西街、南沙河路东段等城市快速路；新改续建五一路、太茅路、东峰路、新旧晋祠路、马练营路、迎泽大街东延、摄乐汾河大桥等道桥项目；改造提升50余条背街小巷。高标准推进地铁2号线建设，积极做好1号、3号线前期工作；坚持地上地下一体规划，同步完成解放路快速化改造。积极创建国家公交都市，优化公交线网布局，新建300个公共自行车站点，努力构建轨道交通、公交车、出租车、公共自行车高效对接的城市公共交通体系。进一步推进太原、晋中同城化，实现规划建设无缝对接。积极拓展地下空间，大力发展静态交通体系，规划建设地下综合管廊，加快建设海绵城市。完成既有建筑节能改造500万平方米。着力提升水、电、气、暖等公共配套设施保障水平，开工建设阳曲、清徐5000吨垃圾焚烧电厂，加快建设餐厨垃圾和污泥处理厂，实现垃圾无害化处理。推进220千伏等24项供电工程建设。继续以城郊森林公园为重点，推进东西北山生态绿色屏障建设，完成造林面积30万亩以上。推进晋阳湖、植物园、汾河三期、迎泽公园等重大园林景观项目建设改造，建成区绿化覆盖率达到41.4%。

坚决打赢城中村改造这场硬仗。加快完成54个城中村拆迁建设安置任务，启动31个城中村拆迁。坚持规划引领，打破乡村界限，集中连片规划，高标准开发建设，做到拆迁与建设并举、安置与开发并重、政府主导与市场化运作有机结合。严控拆迁、成本、资金、市场风险，鼓励和吸引大集团、大企业参与城中村改造。加快推进棚户区改造，启动2万户改造任务，加快在建棚户区安置房建设，力争年内完成晋东、民政园、建材小区等在建工程，确保拆迁群众尽快回迁，加快推进小北关等棚户区改造工程。

以“五城联创”为抓手，全面加强城市管理。增强城市功能，改善人居环境，提升文明程度，为迎接青运会营造良好氛围。构建建管分开、重心下移、区街为主、职责明确、运转有序的城乡管理体制机制。推进智慧城市建设，完善城市公共信息平台，提升网格化、数字化、精细化管理水平。人是城市的主体，人的素质就是城市的素质。要弘扬正能量、培育新风尚，强化规范和文化教化，引导广大市民树立主人翁意识，践行社会主义核心价值观，不断提升文明素养，争当“五城联创”的参与者和推动者。

（三）继续引深“三个突破”，创造率先发展新路径。充分发挥科技创新的引领作用、民营经济的生力军作用、金融振兴的支撑性作用。

要把企业自主创新作为科技创新的基本抓手。坚持需求导向和产业化方向，以企业为主体，充分发挥省城科技资源优势，推进产学研用深度融合，增强科技进步对经济增长的贡献度。深化科技管理体制改革，全力支持山西科技创新城建设。充分发挥开发区和综合保税区的引擎作用，强化科技创新驱动，加快新兴产业发展。全市高新技术企业数量增长10%，高新技术企业销售额占规模以上工业企业的比重提高1个百分点，有效发明专利拥有量增长10%，建设1-3个国家技术创新中心，培育发展10个众创空间和科技企业孵化器，技术合同成交额增长13%。

要把国家小微企业创业创新基地城市示范作为民营经济发展的强大动力，推动“大众创业、万众创新”，打造我市“众创”发展的新形态，激发内生增长的新动力，形成内源发展的新方式。推广新型孵化模式，建设中小微企业创业基地，引导社会力量建设一批低成本、便利化、全要素、开放式创业社区和众创空间，优化创业创新生态，形成线上与线下协同创业创新格局。实行诚信激励政策，推进创业人员和小微企业社会信用体系建设，提高社会诚信度和行为规范性。大力弘扬晋商敢为人先、“无中生有”的创新精神，尊重企业家的社会地位和首创精神，厚植尊重创新、尊重人才的文化“土壤”，激励创新、包容失败，不断释放创业创新的动力和活力。

要把资本市场作为振兴金融的基本载体，加快金融改革创新，提升金融服务水平。大力发展多层次资本市场，力争“新三板”挂牌企业再突破20家，有效利用资本市场扩大直接融资规模。建立政府、银行和企业合作机制，广泛开展与工、农、中、建、交等商业银行的合作，提升金融支持地方经济发展的水平和能力。加强与国开行、农发行等政策性银行的合作，用好用足长周期、低利息的政策红利。完成农村信用社体制改革，增强地方金融发展实力。加快建设互联共享的公共信用信息系统，有效防范、依法处置各类金融风险，打击各类非法集资行为，营造良好金融生态环境。

（四）牢固树立绿色发展理念，持续改善省城环境质量。实行最严格的环境保护制度，全力实施控制燃煤、抑制扬尘、防治尾气、企业提标、整治面源、垃圾秸秆禁烧、治理污水、生态绿化、严格监管、改革创新十大举措，确保省城环境质量全面提升。

全面改善空气质量。完成太古长距离供热输送管网建设，新增供热能力5000万平方米。加快兴能2×66万千瓦、瑞光二期2×35万千瓦热电联供项目建设，为基本实现市区分散燃煤锅炉全替代创造热源条件。实施一电厂关停搬迁，大幅度削减燃煤总量。实施原煤禁烧，推广使用民用洁净焦炭。建立全方位扬尘控制管理系统，实施渣土密闭化清运，全面推行绿色工地标准化管理，重点整治城乡结合部扬尘污染，推进市区裸露地面绿化。淘汰老旧机动车和黄标车2万辆，全面供应国五标准车用汽、柴油，完成纯电动新能源出租车整体更新。调整物流布局，启动中心区各类市场和物流仓储搬迁。推进工业企业提标改造，实施太钢原料场全封闭工程、二电厂铁路运煤和周边环境综合整治工程，完成古交兴能电厂超低排放改造，对焦化等重点行业实施限期提标改造。取缔露天烧烤，中环以内餐饮摊点加装油烟净化装置，城六区内严禁销售燃放烟花爆竹和旺火；杜绝秸秆焚烧，实现全市农田秸秆综合利用全覆盖。全年 PM

2.5年均浓度值下降4%，二级以上优良天气力争增加20天以上，空气质量持续改善，在全国空气质量重点监控城市中排名稳定前移。

加大水环境治理力度。加强饮用水源地保护，建立水源地持续性生态补偿机制，开展汾河水库水源地生态环境治理，强化集中式饮用水源地环境监管，水质达标率稳定保持100%。完成32万吨晋阳污水处理厂一期工程，力争年底开工建设36万吨汾东污水处理厂一期工程，新增污水日处理能力10万吨以上。实施水环境综合整治，完成汾河小店桥上游河道截污等工程。加大黑臭水体整治力度，加快编制建成区17条黑臭水体整治方案，推进城区9条河流治理，建成区黑臭水体比例下降30%。实施再生水利用工程。

（五）推进文化产业文化事业发展，提高国家历史文化名城的影响力。文化是城市的灵魂，是城市竞争力的核心，是城市创新发展的强大动力，影响并决定着城市未来发展的前景和方向。

要以历史文化名城丰富的历史遗存为基础，继续抓好晋祠景区完善提升、明太原县城保护性开发、晋阳古城大遗址保护、太山龙泉寺佛教文化园和青龙古镇保护修复、太化工业文明遗存展示，打造文化旅游大产业、大景区。推进府城文殊寺、普光寺、关帝庙等20余处历史文化遗存抢救保护工程。坚持点面结合，积少成多，久久为功，蔚成大观。以文化战略眼光，处理好历史与现代、继承与发展、保护与创新的关系，留住历史的记忆，彰显文化的神韵，标识名城的高度。

要优化文化产业结构，着力培育新型文化业态和特色文化产业，建设华夏文明主题公园，扩大万达影城的影响力，打造文化领军企业和知名文化品牌。加快文化改革发展步伐，推动文化繁荣，搞好选题策划和资源统筹，聚焦现实题材和重大主题，弘扬和传承晋剧等优秀传统剧种，充分运用多种艺术表现形式，努力打造有历史厚重感和思想感染力的精品佳作。坚持政府主导、社会参与、重心下移、共建共享，推动基本公共文化服务标准化、均等化发展，提高服务效能和普惠水平。加快市图书馆改扩建工程、博物馆布展陈列工程，力争上半年具备对外开放条件。积极开展“文化精品惠民基层行”“书香太原”全民阅读系列活动，不断满足群众精神文化需求。

（六）着力发展和改善民生，不断提升公共服务能力和水平。问题是时代的声音，民生是最大的政治。要把群众的呼声当方向、百姓的期盼当目标，坚持从群众最关切的问题抓起，从群众最希望的实事做起。

持续推进一批重大民生项目。加快幼师、五中、成成中学、一外、二外等新校区建设，积极推进职教园区建设，完善太原学院办学条件，提升教育基础设施建设水平和教育质量。大力支持山西大学东山校区建设，提升省城高等教育的影响力。推进市中心医院、市人民医院、市妇幼医院和省人民医院、省妇幼医院等新院建设，放大优质医疗卫生资源服务效应。加快推进儿童福利院和老年福利院建设，完善社会救助服务政策和体系。大力发展机构养老、社区养老、日间照料中心等多层次养老服务业，落实各项优惠政策，在全社会形成发展养老服务业的强大合力。新建市体育训练中心等一批群众性文化体育活动场馆，加快推进青运会赛事及配套工程，提前做好各项准备工作。

完善创业就业优惠政策，突出做好高校毕业生等重点群体的就业工作，多措并举对困难人员、市属国有破产改制拆迁企业人员实施就业援助。持续扩大社保覆盖面，推进城乡社会保险一体化。全面实施城乡居民大病保险制度，新农合人均筹资标准提高到500元，住院补偿最高支付限额提高到18万元，门诊慢性病补偿比例提高10个百分点。实施公办小学免费托管服务、为义务教育阶段学生办理综合保险等教育惠民事项。

牢固树立安全发展理念。进一步增强红线意识、忧患意识、责任意识，切实落实企业主体责任、部门监管责任、党委和政府领导责任。加强安全预防和隐患排查，重点开展煤矿、非煤矿山、危险化学品、道路交通、公共场所等领域的安全监管和专项整治，有效防范、坚决遏制重特大事故。落实“四个最严”要求，严格食品药品安全监管，规范食品药品生产流通秩序。加强社会治安综合治理，推进社会治安防控体系建设，严厉打击各类违法犯罪，从严整治治安突出问题，维护社会公平正义，保障社会和谐稳定。

（七）强力开展脱贫攻坚，协调推进小康社会目标实现。要树立科学治贫、精准扶贫、根本脱贫理念，坚决打赢三年脱贫攻坚战，今年要实现脱贫2万人。

实施精准扶贫。大力发展农产品加工、物流配送、休闲观光农业、光伏产业、食醋产业等特色产业，强化宝迪、润恒、九牛牧业、太原老陈醋和百企千村等龙头企业的辐射带动功能，激发内生动力，走产业化、市场化扶贫的路子。按照搬得出、稳得住、能致富原则，落实易地搬迁政策，促进移民脱贫。倾斜支持贫困地区发展基础教育、特别是职业教育，让贫困家庭的孩子接受良好教育，掌握一技之长，阻断贫困代际传递。对完全或部分丧失劳动能力的贫困人口，实行社会保障兜底脱贫。探索生态脱贫新路子，通过生态建设与修复实现脱贫。

开展对口帮扶。各城区、开发区及所属行政、企事业单位要对口精准帮扶娄烦、阳曲两县贫困村集中的15个贫困乡、157个贫困村。要立足当地资源优势，重点帮助发展管长远、有效益、能致富的产业，在改善交通、通讯、电力、农田水利等方面多下功夫。各部门要落实对口精准帮扶责任，制定有效推进措施。各行各业要肩负起行业帮扶责任，发挥行业优势，创新帮扶举措，提高扶贫实效。将脱贫攻坚列入年度目标责任考核体系，强化目标考核。

强化主体责任。娄烦、阳曲两县要把脱贫攻坚作为重大机遇，用好用足国家、省、市有关政策，统筹规划，整体推进，树立脱贫致富信心，增强“造血”功能，大力发展县域经济，形成县有龙头企业、乡有特色产业、村有合作组织、户有增收项目的扶贫格局。

（八）加强政府自身改革和建设，提升为民服务能力。认真落实“三严三实”，把忠诚干净担当铭刻在思想上，体现在责任上，落实在行动上。

深化“六权治本”，大力推进“两清单、两张图、两平台”建设，制度的“笼子”要扎得更密，执纪的“利剑”要始终高悬，守纪的“扣子”要扣得更紧，把政府各项工作纳入制度化轨道。自觉接受人大及其常委会的法律监督和工作监督，主动接受人民政协的民主监督，加强行政监察和审计监督，重视社会舆论和社会监督，全面接受人民监督。推进协商民主，加强与人大代表、政协委员以及各民主党派、工商联、无党派人士多渠道沟通协商，完善人大意见建议和政协提案的办理联系机制，提高办理质量和落实率，充分体现人民意愿。

持续推进简政放权、放管结合、优化服务，释放更多改革“红利”。不断深化行政审批制度改革，营造审批事项少、行政效率高、行政成本低、行政过程公开透明的政务环境。推行“多证合一”和“一证多用”，继续推进商事制度、城市管理体制、农村土地制度、国有企业、科技管理体制、金融服务创新等各类改革，以改革推动劳动力、土地和资本等要素资源优化配置，激发创造和发展活力。

认真履行“一岗双责”，落实全面从严治党责任。坚持党风廉政建设和经济社会发展统一部署、同步落实，严格控制“三公”经费，严肃财经纪律，严格公共资金、公共资源、国有资产监管，严厉查处违纪违法案件，始终保持惩治腐败、狠刹四风、打黑除恶“三个高压态势”。自觉遵守廉洁自律准则，严把廉洁从政关口。

深入开展“学党章党规、学系列讲话，做合格党员”学习教育，牢固树立政治意识、大局意识、核心意识、看齐意识。深入学习习近平总书记系列重要讲话精神，认真学习以习近平同志为总书记的党中央领导集体的优良作风，自觉、主动、经常向党中央和习近平总书记看齐。认真学习以王儒林书记为班长的新的省委治晋兴晋强晋的政治担当和工作作风。坚持把以上率下作为基本工作方法，以实际行动一级做给一级看，一级带动一级干，做到胸中有数、落实有策、行动有力，努力创造经得起实践、历史和群众检验的业绩。

各位代表，新起点开启新征程，新目标赋予新使命，新作为绘就新梦想。让我们紧密团结在以习近平同志为总书记的党中央周围，在省委、省政府和市委的坚强领导下，以敢于担当、积极作为的精神状态，抓铁有痕、踏石留印的务实作风，真抓实干、当好表率的拼搏意志，为实现“十三五”良好开局努力奋斗！
